# The Hidden Everyday-Life Burden of Pediatric Type 1 Diabetes: A Household Implementation Framework for Modern Diabetes Technology and Family Care

**DOI:** 10.1007/s11892-026-01643-4

**Published:** 2026-09-05

**Authors:** Zsanett Tesch, Norbert Buzás

**Affiliations:** 1 Department of Health Economics, Albert Szent-Györgyi Medical School, University of Szeged, 35. Kossuth L. sgt., Szeged, H-6724 Hungary; 2 Department of Theoretical Health Sciences and Health Management, Faculty of Health Sciences and Social Studies, University of Szeged, Szeged, Hungary

**Keywords:** Pediatric type 1 diabetes, Household implementation burden, Parental burden, Diabetes technology, School diabetes care, Digital infrastructure, Digital health literacy

## Abstract

**Purpose of Review:**

Frameworks that classify the financial burdens of managing pediatric type 1 diabetes (T1D) primarily focus on measurable material hardship, affordability, and cost-related distress and therefore do not sufficiently detail the household-level resources that care requires from families. This study is a conceptual narrative review that aims to integrate the missing elements into a new three-level burden model, thereby linking the costs visible in clinical care, the household implementation burden borne by families, and the lived everyday experience of the child-parent-family triad. The proposed contribution of the model is to interpret the sustainability of modern T1D care not exclusively in terms of technology access, reimbursement, or clinical effectiveness, but also as a function of families’ temporal, digital, organizational, and decision-making capacity in relation to everyday self-management, educational-setting coordination, caregiver time investment, digital health literacy, and the digital divide.

**Recent Findings:**

The literature documents direct healthcare costs, out-of-pocket expenses, informal care, dietary adaptation, technology-related costs, parental distress, and time burden well when considered separately. However, these elements often do not appear as an integrated, cumulative household implementation burden. In pediatric T1D, the actual usability and sustainability of technology depend not only on device availability and reimbursement but also on the family’s organizational, digital, temporal, and decision-making capacity.

**Summary:**

The T1D-ELB-P framework interprets the burdens placed on families as an organizing principle: it does not merely list domains but shows how financial, digital, temporal, decision-making, and institutional requirements can accumulate as household implementation burdens. The proposed Type 1 Diabetes Everyday-Life Burden-Pediatric framework (T1D-ELB-P) conceptualizes the everyday-life economic burden of pediatric type 1 diabetes as a complementary dimension interpretable at the household and caregiver levels. It highlights five interrelated domains: lifestyle and dietary adaptation; non-reimbursed or partially reimbursed consumables and accessories; digital infrastructure and digital health literacy; time, attention, and decision burden; and educational, extracurricular, and workplace coordination. In this model, technology is not a separate focus but a cross-cutting factor that makes the implementation work borne by families visible across several domains. The clinical and health-policy significance of the framework lies in its highlighting of how the real-world use of modern T1D care is embedded in the everyday functioning of the child-parent-family triad. Future cost-of-illness, health technology assessment, and patient-centered studies would benefit from explicitly measuring family implementation costs and capacities.

**Supplementary Information:**

The online version contains supplementary material available at https://doi.org/10.1007/s11892-026-01643-4.

## Introduction: The Hidden Household Burdens of Modern Pediatric T1D Care

Pediatric type 1 diabetes (T1D) care has changed substantially over recent decades owing to technological development [1–3] and a shift in perspective that supports the social acceptance, school participation, and social integration of affected children [4–6]. Continuous glucose monitoring, insulin pumps, automated insulin delivery systems, data-sharing applications, and remote parental monitoring have become central to everyday T1D management [7]. These innovations may improve glycemic control, increase safety, and reduce certain acute risks [8–10]. As a result, with support from the family, educational institutions, and the broader social environment, affected children can participate more fully in age-appropriate community, kindergarten, school, sports, and leisure activities [11, 12]. On the one hand, this lifestyle shift also creates new types of family and household resource requirements [13, 14]; on the other hand, the everyday operation of modern technology itself requires family resources that partly arise outside clinical care, in the household’s everyday functioning [9, 15].

Traditional health-economic approaches typically describe the burden of disease across the dimensions of direct healthcare costs, indirect costs, and intangible burdens [16, 17]. These categories are essential, but in pediatric T1D they do not fully capture the informal care work families perform, which is fundamental to maintaining safe diabetes care [14, 17, 18]. A cost study focused on pediatric T1D also identified informal family caregiving as a major component of the societal burden of disease [19].

The emergence of modern diabetes technologies has further complicated the picture by creating additional resource requirements that affect family decision-making, affordability, and everyday use [20–22]. These resources are only partially related to the cost burdens discussed in the literature on financial toxicity and affordability [23], and they cannot be fully captured by those concepts. Costs arise not only as countable items but also as time, attention, organization, digital access, and decision-making responsibility. This manuscript interprets these as a household implementation burden and positions them as a distinct layer between clinical costs and lived family experiences, thereby creating the Type 1 Diabetes Everyday-Life Burden-Pediatric framework (T1D-ELB-P). This framework serves not only to describe the family burdens of pediatric T1D but also as an organizing principle that shows how modern T1D care becomes practically feasible, sustainable, or vulnerable through the everyday functioning of the household.

## Review Strategy

This article is a conceptual narrative review. Its aim is to integrate the currently fragmented cost and burden components related to pediatric T1D into a conceptual framework with a parental and household focus. The review draws on the literature on pediatric T1D, diabetes technology, out-of-pocket expenses, informal care, parental distress, school diabetes care, time burden, and health-economic evaluation. The recent technology literature provides the context, but the object of analysis is the household implementation burden and its measurability.

The aim of the conceptual synthesis is to clarify what types of resources should be measured independently in future empirical, health-economic, and patient-centered studies. Accordingly, the manuscript does not seek to test an existing general disease-burden model but to develop a T1D-specific perspective on family implementation.

## Results of the Conceptual Synthesis

### The Child-parent-family Triad as the Unit of Analysis

In pediatric T1D, the unit of analysis cannot be limited to the child living with diabetes alone [18, 24]. A substantial share of insulin administration supervision, glucose monitoring, meal decisions, hypoglycemia prevention, nighttime checks, school or kindergarten coordination, and emergency readiness falls to parents or caregivers [15]. In this context, the parent is not an external observer of the disease burden but a primary executor and coordinator of everyday diabetes management. The central importance of the parental role is further underscored by the fact that, in pediatric T1D, adequate disease management depends to a considerable extent on parents’ competence, efficacy, and everyday caregiving decisions [25]. Parental sleep interruption, constant vigilance, fear of hypoglycemia, workplace adaptation, institutional negotiations, and device logistics directly affect household functioning and indirectly affect the child’s therapeutic safety [24, 26]. In this sense, the family is not merely the context of the child’s care but the operational environment and resource base of disease management: maintaining therapy affects the family’s time management, division of tasks between parents, attention available for siblings, eating and sleeping routines, the daily schedule, and financial and emotional capacities. Therefore, rather than the child-parent dyad, it is justified to treat the child-parent-family triad as the unit of analysis. The clinical significance of the triad perspective is that a lack or exhaustion of family resources is not merely a quality-of-life problem but may also affect continuous technology use, alarm management, adherence, glycemic safety, school participation, and inequalities in care.

### The Three-level Conceptual Framework of T1D-ELB-P

Cost-of-illness analyses and health technology assessments generally capture directly identifiable costs well, such as expenditures for care, pharmacological treatment, and therapeutic devices [27, 28], as well as certain indirect costs, such as productivity loss or informal care [29]. However, they do not account for the household burdens of maintaining treatment in everyday life. These burdens can be defined as the totality of regular or recurring household-level resources required to maintain safe everyday life with T1D. These resources become structurally embedded in daily routines and appear in fragmented form in the literature, so their cumulative effect and household-level organization remain hidden.

**Fig. 1:**
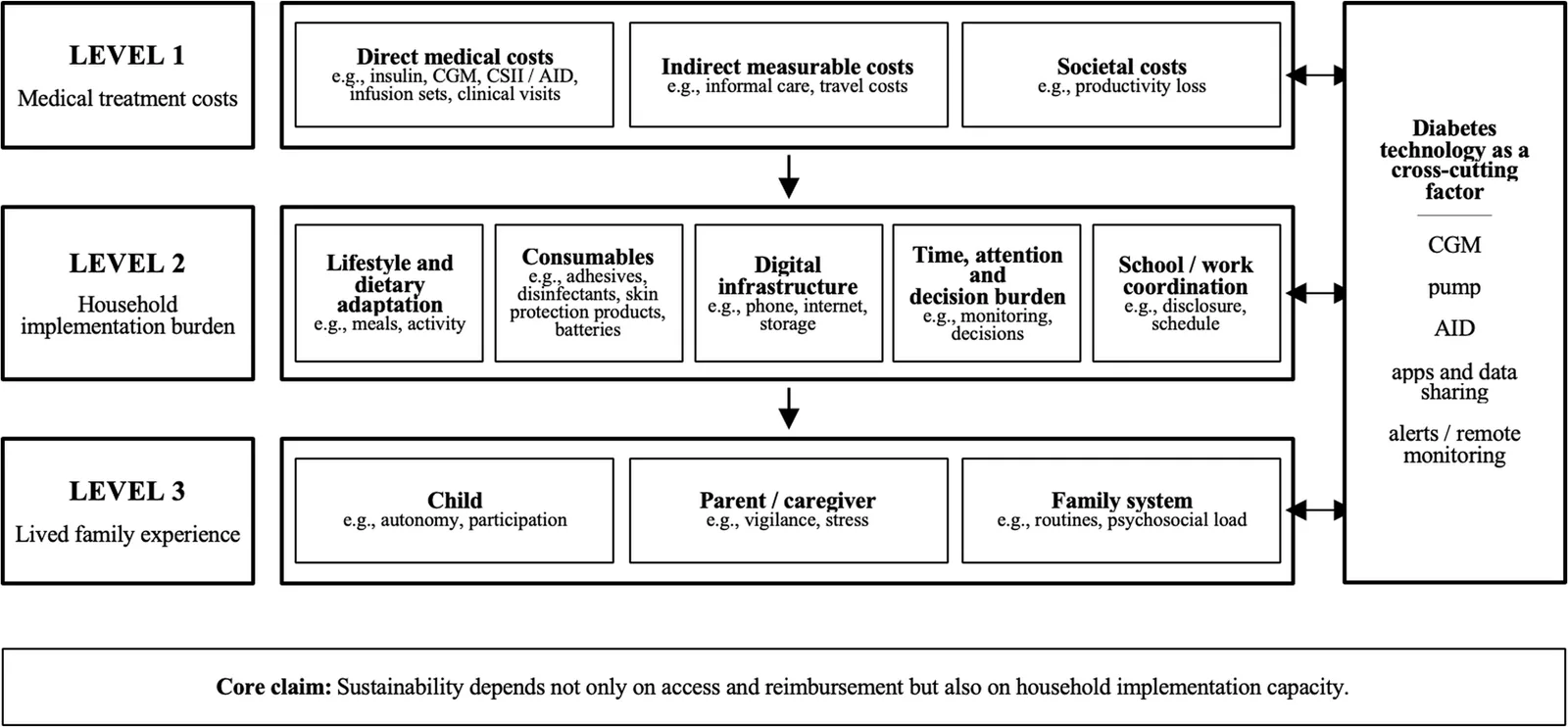
The three levels of burden in the T1D-ELB-P framework

T1D-ELB-P does not replace the measurement of clinical outcomes, quality of life, diabetes distress, or financial toxicity; rather, it adds a complementary analytical layer to these measures. In this sense, the framework is not merely a descriptive taxonomy but an integrating organizing principle: it interprets out-of-pocket expenses, informal care, digital access, school coordination, and parental distress not as separate burden categories but as interrelated implementation conditions. The model (see Fig. 1) distinguishes three levels of burden. The first level includes the previously mentioned costs that are usually visible in the clinical and financing system. The second level describes the household implementation inputs required to deliver care in everyday life. These include the additional costs of lifestyle and dietary adaptation; non-reimbursed consumables and accessories; inputs for maintaining digital and technological infrastructure; time, attention, and decision-making burden; and resource use associated with educational and workplace coordination. These elements do not necessarily appear as formal healthcare expenditures, yet they regularly and cumulatively burden families. The third level shows how these economic, cognitive, and organizational requirements become embedded in the lived experience of the child, parents, and family. This is the context in which household implementation burdens actually affect everyday life.

Therapeutic knowledge, adaptive capacity, lifestyle organization, the transformation of family routines, the child’s autonomy, school and social participation, and psychosocial strain jointly determine the extent to which household-level inputs become a persistent burden on everyday life. This explains why, despite identical access to technology or reimbursement eligibility, real-world outcomes may differ depending on the family’s time capacity, digital preparedness, organizational resources, and caregiver availability [8, 13].

The central proposition of the T1D-ELB-P framework is that standard burden models may underestimate the total disease burden of pediatric T1D when they do not account for the household implementation work required to deliver treatment in everyday life. This omission can have health-policy consequences because financing decisions, reimbursement systems, and resource allocations may be based on cost structures that do not fully reflect the burdens families experience. Integrating the economic, cognitive, digital, and coordination demands of everyday life can therefore provide a more complete interpretation of the social and economic impact of pediatric T1D.

### Main Components of the Household Implementation Burden

Table 1 presents the five main domains of the household implementation burden in pediatric type 1 diabetes. Modern technology is not a separate domain; rather, as a horizontal factor affecting several domains, it makes visible how these inputs are organized into a cumulative household implementation burden.

**Table 1:** Domains of the household implementation burden

Domain	Examples in pediatric T1D	Why is it an economic burden?	Measurement implication
Lifestyle and dietary adaptation	meal planning, special or safer food choices, management of school meals	transforms routine household consumption and time scheduling	monthly extra expenditure, planning time, meal-related compromises
Consumables, accessories, and replacement costs	adhesives, skin protection, disinfectants, spare sensor/pump components, protective cases	small, recurring, and often partly invisible expenses	out-of-pocket items, replacement frequency, supply uncertainty
Digital infrastructure and digital health literacy	smartphone, mobile internet, data sharing, app compatibility, updates, device setup and troubleshooting, data interpretation	both access and digital competence are prerequisites for the effective everyday use of technology	digital access, compatibility problems, troubleshooting time, data-tracking burden
Time, attention, and decision burden	alarm management, interpreting trends, nighttime checks, prescription and device logistics	not always monetized, but constitutes real household resource use	daily time investment, nighttime awakenings, lost working time
Educational, extracurricular, and workplace coordination	individual care plan, school nurse/trained staff, teacher and coach communication, school meals, field trips, sports/camps, parental availability	families often compensate for gaps in institutional support across school and activity settings	number of consultations/interventions, hours lost, level of institutional support, participation constraints

#### Lifestyle and Dietary Adaptation

The burden is not only the price difference between ingredients but also the adjustment of family consumption routines—that is, meal timing and social eating situations—to glycemic management [30–32]. Continuously ensuring the availability of appropriate, safe foods and maintaining constant logistical preparedness for hypoglycemic emergencies also require ongoing planning time and cognitive capacity within the household [33–35].

#### Consumables, Accessories, and Replacement Costs

Small recurring out-of-pocket expenses beyond devices covered by health insurance and often invisible to payers, such as skin barriers, adhesive patches, fixators, cases, and batteries, create cumulative financial strain and ongoing procurement logistics [36–39]. Everyday wear-related inconvenience from diabetes management devices [40, 41], as well as the cognitive burden of unexpected device replacements, also falls under this category.

#### Digital Infrastructure and Digital Health Literacy

Modern management tools make digital infrastructure integral to everyday T1D management [42]. Smartphone compatibility, reliable mobile internet, cloud-based remote data sharing, and access to digital information can be prerequisites for effective use of contemporary technologies [43, 44]. In addition, the importance of digital infrastructure extends beyond device use: regular connection to online parent communities may also become part of the informational and supportive environment of everyday diabetes management [45]. However, access alone is insufficient. Parental electronic health literacy and digital competence influence caregivers’ ability to understand digital information, configure and troubleshoot devices, interpret data, and integrate technology into daily decision-making [46]. Lower digital competence can therefore increase learning time, dependence on professional or peer support, troubleshooting effort, and uncertainty, while higher competence may reduce some of these demands. Formal access does not necessarily translate into sustained use: if a family’s digital capital, infrastructure, or literacy is insufficient, a digital divide may persist despite access to technology [46, 47]. Within T1D-ELB-P, digital literacy is therefore treated as a household implementation resource rather than merely an individual skill.

#### Time, Attention, and Decision Burden

Managing the flood of alarms from modern sensors and closed-loop systems (AIDs), continuously interpreting data, and sleep interruptions at night represent non-monetized household resource use that directly limits parents’ daytime work and recovery capacity [48]. Lack of rest may, in turn, affect the quality of next-day decisions and thus the effectiveness of diabetes care [49].

#### Educational, Extracurricular, and Workplace Coordination

Daycare/kindergarten and school are treated within the same implementation domain but are not assumed to be equivalent settings. In daycare and kindergarten, diabetes care typically requires more direct adult supervision, whereas school-aged children may progressively assume greater self-management responsibility; staffing, institutional rules, and available support can also differ across settings [50]. Safe T1D care may require trained personnel, an individual care plan, institutional capacity for insulin administration and glucose monitoring, and continuous communication among parents, the healthcare team, and educational staff [51, 52]. A school nurse, where available, or other specifically trained school personnel can reduce the need for parents to provide direct on-site support. Coordination also extends beyond classroom time to physical education, field trips, school camps, team sports, and other extracurricular activities, where communication with school nurses, teachers, physical education instructors, or coaches and ready access to diabetes supplies are important [53, 54]. If institutional support is incomplete, unequal, or difficult to implement, the burden of coordination and responsibility falls back on the family [50, 55]. Parents may then compensate through continuous availability, direct administration, additional planning, and workplace flexibility, turning coordination into a recurring household resource demand [56, 57].

### Technological Sustainability Beyond Access and Reimbursement

The T1D-ELB-P framework complements assessments of technology beyond access, reimbursement, and clinical effectiveness by incorporating usability and sustainability. When assessing the real-world value of technology, it is also necessary to consider the household resources—digital devices, data connectivity, digital health literacy, time capacity, technical problem-solving, and caregiver availability—required for sustained use. A device’s clinical effectiveness and reimbursement status alone are insufficient to determine whether it can be integrated into daily family life [58], as uptake and continued use are also shaped by physical, usability, cost, healthcare system, access, and psychosocial barriers [59]. Accordingly, the framework considers how costs, time investment, digital conditions, skills, and institutional support may enable or constrain sustained use of technology.

## Discussion

### Relationship to Existing Frameworks: From Fragmented Burdens to Integrated Implementation

Although the socioeconomic and humanistic impacts of pediatric type 1 diabetes (T1D) are well documented, current health-economic models and clinical frameworks (Table 2) remain highly fragmented. The existing literature captures individual dimensions of the challenges associated with diabetes, but it does not provide a conceptual description of how these factors combine into a single household-level operating system.

**Table 2:** T1D-specific financial/burden approaches in the literature

T1D-specific financial burden / burden approach	Focus of the economic/family burden	Limitation of the model/framework	Additional value of T1D-ELB-*P*
Pediatric T1D direct household cost / economic burden [27]	Direct costs: insulin, blood glucose meter, test strips, syringes, HbA1c testing. The study estimated the family cost of consumables needed for minimum care of a child with T1D in 15 lower-income countries.	Cost-of-supplies focus; it does not model household organizational work, time investment, digital technology administration, labor market losses, or lack of family capacity separately.	Interprets direct costs not as isolated expenses but as household implementation resources that determine the feasibility of everyday care.
Pediatric T1D family financial burden / out-of-pocket expenditure [60]	In the financial expenses of families raising a child with T1D, T1D care places a substantial material burden on families (OOPE), mainly because of costs related to SMBG, insulin, and hospital care.	Directly relevant empirical cost-burden study, but not a conceptual family implementation model. It shows less clearly how financial burden is transformed into daily decisions, delays, technology-use compromises, or parental workload.	Makes visible the mediating mechanisms between financial burden and everyday diabetes care practice: what the family omits, simplifies, postpones, or reorganizes.
Pediatric T1D humanistic burden framework [18]	Frames the humanistic burden of pediatric T1D and the burdens of informal caregivers. The article discusses child-, caregiver-, and family-level burden dimensions.	The economic component is absent; it does not elaborate on the relationship among financial pressure, digital technology, labor-market compromises, and household implementation.	Adds an economic-implementation layer alongside humanistic burden: family burden is not only emotional or quality-of-life related, but also a problem of time, money, technology, and work-organization capacity.
Cost-related rationing / access barrier in T1D [28]	Continues investigating out-of-pocket expenses (OoPEs) and rationing of insulin and diabetes supplies, including the impacts of the COVID-19 pandemic, for people with type 1 diabetes (T1D).	Strong access and rationing focus, but mainly concentrates on medication and device use.	Extends the concept of rationing: not only insulin or test strips can be rationed, but also time, attention, digital care capacity, parental presence, and coordination work.
Diabetes financial burden measurement validation / COST-FACIT diabetes adaptation [61]	Examines the reliability and validity of the 11-item COST-FACIT measure in a diabetes population. The authors identified two factors: general financial situation and the impact of disease on financial situation.	Not pediatric T1D-specific: the sample consists of adults with diabetes and high A1C, predominantly T2D, from a single healthcare system.	Financial burden can be further interpreted not only as patient-level distress or a metric, but as a constraint on family functioning: how it affects parental time investment, care coordination, technology use, and daily care decisions.
Pediatric T1D societal cost / informal care cost [19]	Estimates the socioeconomic costs of pediatric T1D in Spain from a societal perspective. The paper emphasizes that pediatric T1D causes substantial societal costs and that informal care is the largest cost category.	Strong societal-cost and informal-care focus, but essentially a cost-estimation study. It provides less detail on how the informal-care burden is organized into daily family work, shared responsibility, technology administration, and parental labor-market adaptation.	Interprets informal care cost not only as a monetized burden, but also as daily care capacity embedded in family functioning: who maintains the child’s T1D care, when, and with what time, attention, and organizational input.
Diabetes financial burden / financial toxicity overview [23]	Reviews the financial burden associated with diabetes globally. It identifies four main mechanisms: direct out-of-pocket costs, indirect costs including productivity loss and caregiver burden, coverage gaps, and system-level structural barriers.	Diabetes-specific review, but not pediatric T1D-specific and not a family household implementation model. Although it mentions caregiver burden and pediatric T1D families, the main analytical level remains general diabetes care / healthcare system / patient population.	Narrows the global financial-burden framework to the family level in pediatric T1D, showing how direct and indirect costs, coverage gaps, and access barriers translate into daily family care organization, technology maintenance, parental time investment, and labor-market adaptation.
Parental occupational and financial consequences [29]	Examines the consequences of a child’s T1D diagnosis for parental employment and family finances. It is especially important for describing maternal labor-market burden.	Labor-market focus, but not a full economic-burden framework. It primarily examines occupational consequences and does not integrate direct costs, technology administration, and daily micro-burdens of care organization in detail.	Treats parental labor-market adaptation not as a separate consequence, but as a central dimension of family T1D care capacity.
Pediatric T1D multidimensional family burden [62]	Examines the financial, social, and psychosocial burdens on families of children with T1D together.	Multidimensional, but small-sample and descriptive; it does not detail the daily implementation mechanisms resulting from financial burden.	Connects financial, social, and psychosocial dimensions in a household care-capacity model.
Pediatric T1D social and financial burden [63]	Examines the social and financial burden of pediatric T1D families; it discusses multiple dimensions of caregiver burden, including financial and emotional burdens.	It links the mechanisms of financial burden only partly with time investment and work, and not at all with technology use and daily caregiving decisions.	Arranges several dimensions of family burden into an operating model: financial pressure, parental time, digital conditions, and care coordination appear as mutually reinforcing factors.
Pediatric / young adult T1D insulin cost-sharing policy [64]	Estimates the expected reduction in insulin out-of-pocket spending for children and young adults under cost-sharing caps.	Mainly focuses on insulin costs. It does not examine other devices, CGM/pump access, monitoring costs, travel, parental time, or labor-market losses.	Interprets insulin access as part of total family care capacity, where obtaining medication is only one element of the financial, temporal, technological, and organizational inputs required to maintain daily care.
T1D access barrier / insulin and supply acquisition [65]	Discusses affordability as well as obtaining, procurement, administrative, and access barriers for insulin and diabetes supplies.	Not pediatric-specific; it focuses mainly on access barriers.	Treats access barriers as part of everyday implementation: what matters is not only whether the device is theoretically available, but also whether the family can regularly obtain, maintain, and use it.
Parental objective burden and time cost in pediatric T1D [66]	Interprets parental burden not only as emotional distress, but also as objective caregiving input. The economic burden appears primarily as a time and availability burden.	Captures parental burden mainly as caregiving input and distress, and less as an operating mechanism organizing the family’s daily care capacity.	Treats parental time investment as a central resource of family care capacity and links it with financial, technological, and organizational burdens.
Parental electronic health literacy and pediatric T1D technology management [46]	Examines parental electronic health literacy in pediatric T1D, especially in relation to digital information sources, sensor use, pump/sensor therapy modalities, and parental diabetes-management attitudes.	Focuses on digital health literacy and disease management, foregrounding individual skills. The economic component appears mainly indirectly: technology use presupposes access, digital competence, parental learning capacity, and time needed for home operation.	T1D-ELB-P interprets digital health literacy not only as an individual skill, but as a family implementation resource: using sensors, pumps, and digital information sources requires financial access, technological maintenance, data tracking, and daily decision support.
Socioeconomic status and diabetes technology use in youth [67]	Links T1D technology use to financing models and socioeconomic status. This is directly relevant because it examines the financial and social conditions of access to digital technologies.	The focus is on inequality in technology access, not on how the family operates the technology in everyday care.	Connects technology access with family operating capacity: what matters is not only whether the family obtains technology, but also whether it has the time, knowledge, digital competence, and stable resources to maintain it.
Parent diabetes distress / Parent Diabetes Distress Scale (PDDS) [68]	Measures diabetes-specific emotional distress in parents, including personal, teen-management, parent-teen relationship, and healthcare-team distress.	Captures emotional appraisal and psychological response to diabetes-management demands; it does not quantify the household resources or implementation work required to perform care.	Complements distress measurement by identifying financial, temporal, digital, organizational, and coordination inputs that may contribute to, coexist with, or be intensified by distress.

### Limitations of Existing Cost-of-illness and Financial-burden Approaches

Traditional health-economic evaluations and studies of specific out-of-pocket expenses [27, 28, 60, 64] primarily focus on quantifiable consumable costs, such as insulin, test strips, and sensors. Although these data are essential for assessing financial toxicity, they treat household expenditures as isolated transactions, obscuring the mechanisms by which financial pressure leads to rationing of daily treatments, implementation delays, or compromises in technology use. Similarly, societal-level cost estimates that identify informal care as the largest economic component [19] do not explain how this care is operationalized in everyday life. Informal care is not a time-loss category that can be simply monetized, but rather the active and continuous allocation of cognitive energy, administrative logistics, and data interpretation.

### Gaps in Humanistic and Occupational Frameworks

On the psychosocial spectrum, multidimensional burden models [18, 62, 66] and inequality models [67] discuss caregiver distress and emotional strain in detail, but they lack an economic-implementation layer. By contrast, occupational studies focus almost exclusively on long-term career consequences and maternal labor-market withdrawal [29], without linking these structural losses to everyday micro-administrative burdens and technological upkeep.

### Relationship to Diabetes Distress and Psychosocial Burden

Diabetes distress is a clinically important but conceptually distinct construct from household implementation burden. The Parent Diabetes Distress Scale (PDDS), for example, identifies personal distress, teen-management distress, parent-teen relationship distress, and healthcare-team distress among parents of adolescents with T1D [68]. Several experiences that contribute to distress overlap with the operational demands represented in T1D-ELB-P, including vigilance, concerns about child self-management, technology management, nighttime disruption, and interactions with healthcare or educational systems. The distinction lies in the level of analysis: diabetes-distress measures primarily capture the emotional appraisal and psychological response to diabetes-related demands, whereas T1D-ELB-P describes the financial, temporal, digital, organizational, and coordination inputs required to perform care. These constructs may influence one another, but they should be measured separately in empirical validation to avoid conflating implementation demands with their emotional consequences.

### The Integrative Novelty of T1D-ELB-P

The T1D-ELB-P framework bridges these analytical gaps by shifting the focus of analysis from isolated outcomes to the household as the operational site of care. Rather than introducing new clinical variables, it integrates existing concepts, such as financial toxicity [23], electronic health literacy [46], and institutional barriers [65], into a single, coherent capacity model (see Fig. 2 in Supplements). In this framework, device acquisition, parental time investment, and digital competence are not separate or peripheral burdens but interdependent operating preconditions. Together, these conditions may shape whether a modern diabetes intervention can be sustained over time or remains vulnerable in real-world settings.

### Digital Health Literacy as a Household Implementation Resource

Digital health literacy can modify family burden even when devices and connectivity are formally available. Parental electronic health literacy has been associated with pediatric T1D disease-management attitudes and technology use [46]. From an implementation perspective, the relevant issue is not only whether a caregiver can access digital information but also whether the household can convert that information into reliable device setup, troubleshooting, data interpretation, remote monitoring, and everyday decisions. Families with limited digital skills may need more training, time, technical assistance, and repeated contact with professionals or peers. Conversely, adequate digital literacy may reduce avoidable troubleshooting and information-search burden. T1D-ELB-P therefore places digital literacy alongside hardware and connectivity within the digital implementation domain.

### Educational Settings and Extracurricular Participation

The distinction between daycare/kindergarten and school is primarily developmental and organizational rather than a separation into different burden domains. Younger children generally require more direct adult execution of diabetes tasks, whereas increasing age can shift portions of monitoring, treatment, and communication toward the child. Nevertheless, families remain dependent on institutional capacity across both settings [50]. School nurses or other trained personnel can serve as an implementation resource by providing or coordinating diabetes care during the school day [53]. The same logic extends to sports and extracurricular activities: safe participation may require anticipatory planning, access to glucose and insulin supplies, and communication among the child, parents, healthcare providers, the school nurse, the physical education instructor, or the team coach [53, 54]. These supports can reduce the need for parents to be physically present and can therefore affect both household time burden and the child’s opportunity for age-appropriate participation.

### Cultural and Linguistic Context

Language and cultural context may modify several T1D-ELB-P domains simultaneously rather than constituting a separate burden domain. In culturally and linguistically diverse families, qualitative evidence has identified dietary challenges, linguistic barriers, limited support, and knowledge as important influences on pediatric T1D management [69]. Limited proficiency in the dominant healthcare or school language may increase the time and cognitive effort required to understand written information, learn to use devices, communicate with educational staff, and navigate services. Cultural practices may also shape dietary adaptation, family role allocation, expectations about child autonomy, disclosure of diabetes, and the acceptability of visible technologies. Conversely, culturally responsive education, appropriate interpreter or translation support, pictorial resources, and culturally concordant communication may reduce implementation burden. These factors are therefore best understood as contextual conditions that can amplify or buffer household demands across multiple domains.

### Equity Implication

Household implementation costs may contribute to social differences in technology use [27]. Even with identical formal eligibility or insurance coverage, families may differ in access to adequate digital devices, digital literacy, flexible work hours, school or school-nurse support, transportation options, caregiver time, and culturally or linguistically appropriate information. Therefore, equity concerns are not only whether the child has access to the necessary therapy or technology but also whether the family has the practical and contextual resources required to deliver treatment safely, continuously, and sustainably in everyday life.

### Health-policy Implications

To translate the theoretical constructs of the T1D-ELB-P framework into practical application and clinical validation, the proposed domains need to be operationalized as standardized, empirically measurable indicators. The methodological limitations of traditional health-economic and psychosocial questionnaires, such as recall bias and the invisibility of hidden micro-inputs, can be addressed with a transdisciplinary measurement matrix (see Table 3 in Supplements), in which indicators are organized not as isolated cost blocks but according to how they influence the feasibility, sustainability, and within-family distribution of everyday care. In practice, this also means considering resources outside the clinic, including school nurses or other trained school personnel, culturally and linguistically appropriate education, and digital literacy support when these resources reduce household implementation work.

## Limitations of the Review and Proposed Framework

This article is a conceptual narrative review rather than a systematic review. The literature was selected to support conceptual integration across health-economic, psychosocial, technological, and educational domains; therefore, the review does not claim exhaustive coverage and does not include a formal study-quality appraisal. The underlying evidence is heterogeneous with respect to child age, diabetes duration, healthcare financing, school systems, technology availability, socioeconomic context, language, and culture, which limits direct transferability of individual findings across settings. Diabetes technologies and digital ecosystems evolve rapidly, so specific device or digital requirements may change over time. T1D-ELB-P should therefore be regarded as a hypothesis-generating and measurement-oriented framework that requires prospective empirical validation before it is used to quantify burden or guide resource-allocation decisions.

## Future Research Directions

Future research should develop and validate standardized, T1D-specific measures to assess the burdens of everyday household implementation. These measures should distinguish recurring maintenance resources from clinical outcomes, general quality of life, diabetes distress, and financial crises while testing how these constructs interact. Longitudinal and mixed-methods studies are needed to examine how small recurring costs, time inputs, digital literacy, nighttime vigilance, educational and extracurricular coordination, and language/cultural context accumulate and relate to household income, parental work, distress, child age and autonomy, institutional support, technology persistence, and clinical outcomes. Family cohorts spanning multiple countries or healthcare systems would be particularly useful for examining how reimbursement, school-nurse or trained-staff availability, digital infrastructure, culturally and linguistically appropriate support, and labor-market flexibility modify everyday implementation burdens.

## Conclusions

The economic consequences of pediatric T1D extend beyond direct healthcare expenditures and episodes of overt financial distress. In the era of modern diabetes technologies and data-driven self-management, day-to-day disease management depends substantially on family implementation resources. The proposed T1D-ELB-P framework conceptualizes the recurring, distributed, and household-level resources required to maintain safe T1D management for a child. The real-world usefulness of technological innovations depends not only on device effectiveness or reimbursement, but also on whether families can sustain the temporal, digital, cognitive, organizational, and institutional work required for everyday use. For children and their families, technology is most useful when it can be integrated into school, extracurricular, workplace, family, and social routines. The proposed contribution of T1D-ELB-P is to interpret lifestyle and dietary adaptation, accessories and consumables, digital infrastructure and literacy, time and decision burden, and educational/extracurricular/workplace coordination as implementation conditions rather than peripheral inconveniences. Cultural and linguistic context, developmental stage, diabetes distress, and institutional support may further modify these demands. Empirical validation is needed before the framework’s domains can be weighted, aggregated into a summary measure, or used to guide resource allocation decisions.

## Key References

- Allen V, Mahieu A, Kasireddy E, Shouman W, Pourrahmat MM, Collet JP, et al. Humanistic burden of pediatric type 1 diabetes on children and informal caregivers: systematic literature reviews. Diabetol. Metab. Syndr. BioMed Central Ltd; 2024. https://doi.org/10.1186/s13098-024-01310-2.

◦ This is the most direct contextual reference for understanding the burden of disease at the child–caregiver–family level. The manuscript goes a step further by incorporating economic, temporal, digital, and organizational implementation layers alongside the humanistic burdens.

- Azimi T, Johnson J, Campbell SM, Montesanti S. Caregiver burden among parents of children with type 1 diabetes: A qualitative scoping review. Heliyon. Elsevier Ltd; 2024;10. https://doi.org/10.1016/j.heliyon.2024.e27539.

◦ It directly summarizes the literature on parental/caregiver burden and provides strong support for the manuscript’s central argument that managing a child with T1D requires sustained family caregiving, organizational, and emotional effort.

- Hölgyesi Á, Luczay A, Tóth-Heyn P, Muzslay E, Világos E, Szabó AJ, et al. The Impact of Parental Electronic Health Literacy on Disease Management and Outcomes in Pediatric Type 1 Diabetes Mellitus: Cross-Sectional Clinical Study. JMIR Pediatr Parent. JMIR Publications Inc.; 2024;7. https://doi.org/10.2196/54807.

◦ This reference interprets the use of modern T1D technology not merely as access to devices but as a process linked to parental digital literacy and day-to-day management. The paper reinforces the arguments regarding digital infrastructure, e-health literacy, and technological sustainability.

- Howard V, Maguire R, De Bruin E, Deane-King J, Duda N, Corrigan S. Around-the-clock: Caregiving at night for juveniles living with type 1 diabetes–a systematic review. Psychol Health Med. Routledge; 2025;30:1701–22. https://doi.org/10.1080/13548506.2025.2468529.

◦ It specifically documents the nonstop, round-the-clock burden of care and parental on-call responsibilities, which closely align with the “time, attention, and decision burden” dimension of the T1D-ELB-P model. It is particularly important because it provides systematic evidence for the real, non-monetized burden of nocturnal caregiving—a dimension that the T1D-ELB-P framework explicitly incorporates as part of the household implementation burden.

## Supplementary Information

Below is the link to the electronic supplementary material.

Supplementary Material 1

## Data Availability

No datasets were generated or analysed during the current study.

## Declarations

### Human/Animal Studies

This article does not contain any studies with human or animal subjects performed by any of the authors.
