# Supplementary material for: The Hidden Everyday-Life Burden of Pediatric Type 1 Diabetes: A Household Implementation Framework for Modern Diabetes Technology and Family Care
Source: Curr Diab Rep. 2026 Sep 5;26(1):26. doi: 10.1007/s11892-026-01643-4 (PMC13546161; doi:10.1007/s11892-026-01643-4)
Supplement: Supplementary file 1 [file 11892_2026_1643_MOESM1_ESM.docx]

**Supplements**

**Figure 2** How does the T1D-ELB-P framework add value?

**Table 3** Validation of the T1D-ELB-P framework

| **Measurement area** | **Indicators** | **Related outcome** |
| --- | --- | --- |
| Out-of-pocket expenses | How much did the family spend on accessories, replacements, and digital conditions? | household cost burden |
| Time burden | How much time did T1D management and device logistics require per day? | parental burden, lost work time |
| Nighttime readiness | How many times was sleep interrupted for diabetes-related reasons? | parental exhaustion, distress |
| Educational and extracurricular coordination | How many consultations, parental interventions, or activity-specific plans were required at school, sports, camps, or field trips? Was a school nurse or trained staff member available? | school/social participation, family organizational burden, lost work time |
| Digital infrastructure and literacy | Were there compatibility, data-connection, app-management, setup, troubleshooting, or data-interpretation problems? | sustainability of technology use, technical/cognitive burden |
| Language and cultural context | Did language barriers, translation needs, culturally specific dietary practices, or culturally adapted education increase or reduce the work required to manage T1D? | communication burden, equitable implementation, educational access |
